# P-551. The Fight Against Antimicrobial Resistance: Importance of Ongoing Investment, with Projections for Lives and Healthcare Dollars Saved

**DOI:** 10.1093/ofid/ofaf695.766

**Published:** 2026-01-11

**Authors:** Alex Belote, Kellie Wark

**Affiliations:** University of Kansas Medical Center, Kansas City, Kansas; University of Kansas, Kansas City, Kansas

## Abstract

**Background:**

Antimicrobial Resistance (AR) is now the leading cause of death worldwide and forecasted to cost $3.4 trillion/year by 2030. While COVID pushed the limits of the healthcare system, 80% of hospitalized COVID patients received antibiotics, many unnecessarily, and AR infections increased by 18-20%. AR contributed to an excess of 14.9 million deaths, including millions in the Americas. The CDC (Centers for Disease Control) allocated over $165 million in 2023 to fight AR. Ongoing measures, with bipartisan support, are needed to help mitigate these threats.

**Table 1.**
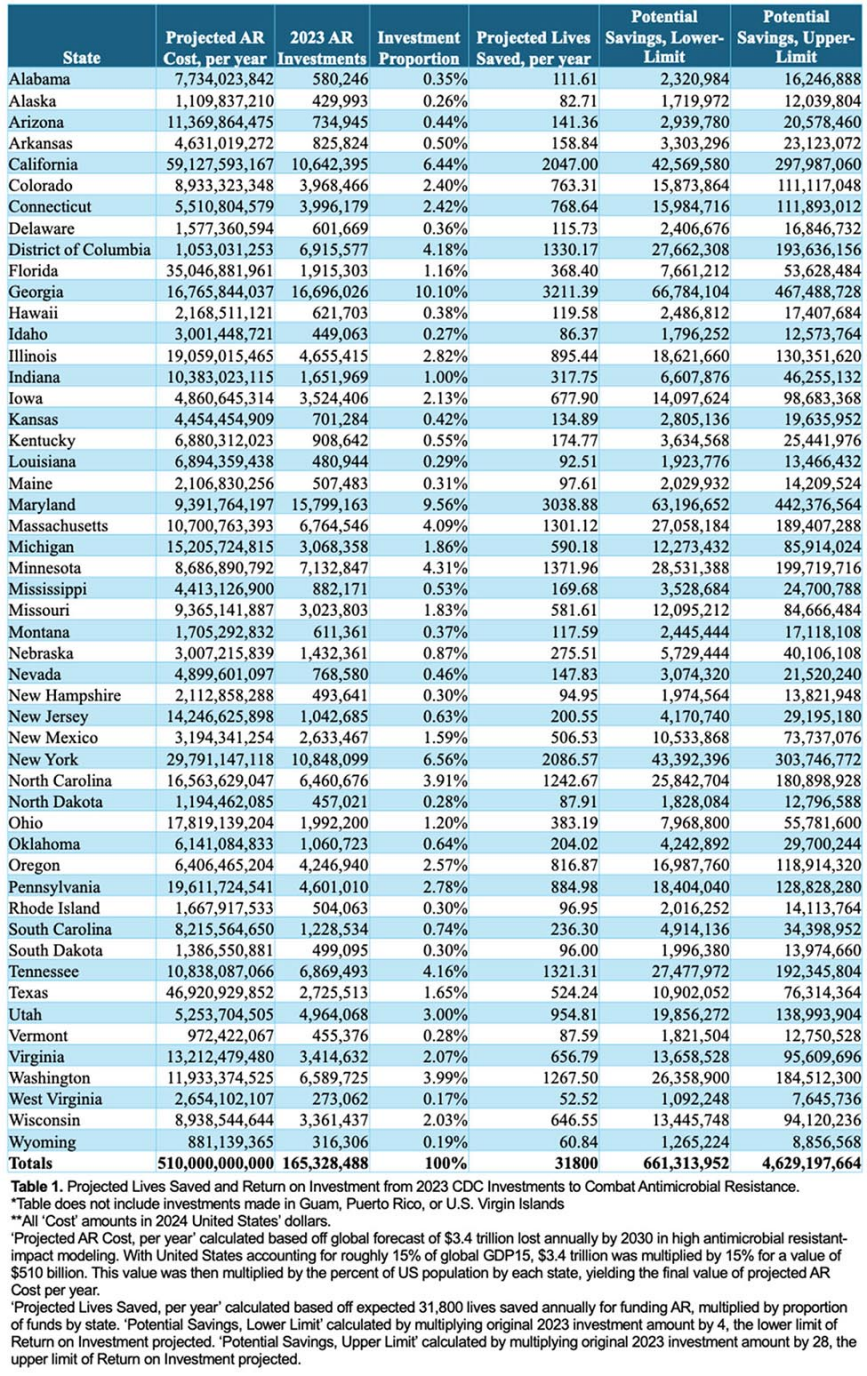
Projected Lives Saved and Return on Investment from 2023 CDC Investments to Combat Antimicrobial Resistance

**Methods:**

2023 AR Investment figures were obtained from the CDC. “Projected AR Cost” calculated based off projections for $3.4 trillion lost/year. The U.S accounts for ∼15% of global GDP and was applied to $3.4 trillion, yielding $510 billion lost in the U.S. Final state values were applied to $510 billion based off population proportions. ‘Projected Lives Saved’ was calculated based on models forecasting 20,000-43,600 lives saved/year by AR funding, averaged to 31,800 lives saved/year. This value was multiplied by proportion of funds by state to calculate final values. ‘Potential Savings, Lower Limit’ based from original 2023 investment amount multiplied by 4, the lower limit of return on investment (ROI) projected. ‘Potential Savings, Upper Limit’ calculated by multiplying by 28, the upper limit of ROI.

**Results:**

Table 1 outlines projections of cost, lives saved, and ROI related to AR by state and national totals. Densely populated states like California could see costs from AR as high as $59 billion. Distributions from the $165 million CDC investment are shown. The $165 million invested in 2023 could see a national ROI of over $661 million, and as high as $4.63 billion. Kansas, with only 0.42% of the national funding to combat AR at $701k, could be expected to see between $2.8-19.6 million saved over 10 years. 31,800 lives are also projected to be saved per year from this investment. Despite minimal funding to combat AR, the less populated states have scores of projected lives saved. Georgia and states with higher funding are projected to prevent >3,000 deaths/year.

**Conclusion:**

AR is projected to cost millions of lives and billions of dollars and in years to come. Ongoing funding will be essential to help curb the spread of AR and save lives.

**Disclosures:**

All Authors: No reported disclosures

